# Health-related quality of life and chronic obstructive pulmonary disease in early stages – longitudinal results from the population-based KORA cohort in a working age population

**DOI:** 10.1186/1471-2466-14-134

**Published:** 2014-08-09

**Authors:** Margarethe E Wacker, Matthias Hunger, Stefan Karrasch, Joachim Heinrich, Annette Peters, Holger Schulz, Rolf Holle

**Affiliations:** 1 Institute of Health Economics and Health Care Management, Helmholtz Zentrum München (GmbH) - German Research Center for Environmental Health, German Center for Lung Research, Comprehensive Pneumology Center Munich (CPC-M), Ingolstaedter Landstr. 1, 85764 Neuherberg, Germany; 2 Institute of Epidemiology I, Helmholtz Zentrum München (GmbH) - German Research Center for Environmental Health, German Center for Lung Research, Comprehensive Pneumology Center Munich (CPC-M), Ingolstaedter Landstr. 1, 85764 Neuherberg, Germany; 3 Institute and Outpatient Clinic for Occupational, Social and Environmental Medicine, Ludwig-Maximilians-Universität München, Ziemssenstr. 1, 80336 Munich, Germany; 4 Institute of General Practice, University Hospital Klinikum rechts der Isar, Technische Universität München, Orleansstr. 47, 81667 Munich, Germany; 5 Institute of Epidemiology II, Helmholtz Zentrum München (GmbH), German Research Center for Environmental Health, Ingolstaedter Landstr. 1, 85764 Neuherberg, Germany

**Keywords:** COPD, Health-related quality of life, SF-12, Comorbidities, General population study, Longitudinal

## Abstract

**Background:**

It is widely recognized that health-related quality of life (HRQL) is impaired in patients with Chronic Obstructive Pulmonary Disease (COPD), but there is a lack of research on longitudinal associations of COPD and HRQL. This study examined the effects of COPD in early stages of disease on HRQL over ten years in a working-age general population setting in Southern Germany while considering the influence of common comorbidities.

**Methods:**

In the population-based KORA F4 study (2006–08) 1,321 participants aged 41–61 years performed spirometry and reported information on HRQL (measured by the generic SF-12) and comorbidities. For the same participants, HRQL information was available seven years before and three years after the lung function test from the previous S4 (1999–2001) and the F4L follow-up study (2010). Using linear mixed models, the physical and mental component summary scores (PCS-12 / MCS-12) of the SF-12 were compared over time between COPD groups.

**Results:**

7.8% of participants were classified as having COPD (according to the LLN definition and the Global Lungs Initiative), 59.4% of them in grade 1. Regression models showed a negative cross-sectional association of COPD grade 2+ with PCS-12 which persisted when comorbidities were considered. Adjusted mean PCS-12 scores for the COPD grade 2+ group were reduced (−3.5 (p = 0.008) in F4, −3.3 (p = 0.014) in S4 and −4.7 (p = 0.003) in F4L) compared to the group without airflow limitation. The size of the COPD effect in grade 2+ was similar to the effect of myocardial infarction and cancer. Over ten years, a small decline in PCS-12 was observed in all groups. This decline was larger in participants with COPD grade 2+, but insignificant. Regarding MCS-12, no significant cross-sectional or longitudinal associations with COPD were found.

**Conclusion:**

Despite small HRQL differences between COPD patients in early disease stages and controls and small changes over ten years, our results indicate that it is important to prevent subjects with airflow limitation from progression to higher grades. Awareness of HRQL impairments in early stages is important for offering early interventions in order to maintain high HRQL in COPD patients.

## Background

Chronic obstructive pulmonary disease (COPD) is a leading cause of morbidity and mortality worldwide. As it is projected to be the fourth leading cause of death in 2030 [1] COPD is a major global public health problem and responsible for increased healthcare utilization and costs [2].

The heterogeneous disease, which in high- and middle-income countries is mainly caused by tobacco smoking, is characterized by an airflow limitation which is not fully reversible, usually progressive and accompanied by a chronic cough, sputum production and dyspnea. Furthermore, acute exacerbations contribute to the overall severity of disease as these episodes are accompanied by worsened symptoms and associated with increased decline in lung function. Comorbid conditions and systemic or extrapulmonary consequences are frequent in COPD patients, particularly at higher grades, and their importance has been increasingly recognized in recent years. Cardiovascular, metabolic, mental, musculoskeletal disorders and cancer are the most common comorbidities and influence disease outcomes [3,4].

Characteristics of COPD and its concomitant phenomena impose a burden on patients. Health-related quality of life (HRQL) as an important patient-reported outcome measure in COPD has received increased interest in the recent past. A number of studies have examined health-related quality of life in COPD patients in stable disease or during acute exacerbations using both generic and disease-specific instruments and tried to quantify the impact of the disease on patients [5].

While disease-specific instruments such as the St George’s Respiratory Questionnaire (SGRQ) [6,7] are designed to capture the specific aspects associated with a particular disease, generic instruments such as the Short Form 12 Health Survey Questionnaire (SF-12) measure overall health states. Therefore, they are more suitable to assess the impact of comorbid conditions, and they allow comparisons across patients with different diseases and with the general population.

Many of the studies employing generic instruments such as the SF-12 or the EuroQol 5 dimension (EQ-5D) only include patients with moderate or severe COPD [8-10]. However, mild COPD is the most frequent stage of the disease, with a prevalence of 7.4% in Germany in those aged 40 and above, while the prevalence of COPD grades 2 and 3 is estimated to be 5.0% and 0.8% respectively [11].

Only few studies have compared HRQL in COPD patients with values in healthy controls and included early disease stages which are often undiagnosed. A recent cross-sectional analysis based on data from the international Burden of Obstructive Lung Disease (BOLD) study found that subjects with COPD had lower physical and mental HRQL scores than subjects without COPD [12]. It also showed an increasing negative impact on health status with increasing disease severity. Subjects with grade 1 COPD had a similar health status to subjects without airflow limitation.

There is a lack of studies investigating the longitudinal impact of COPD on HRQL while considering comorbidities which also impact health status. Most studies focus on HRQL changes after pharmacological and non-pharmacological interventions in selected patients groups [13-15].

Since information about the progression of HRQL in early stages of COPD is scarce, the aim of this study was to compare HRQL development over a ten year period in patients with early COPD stages and in healthy controls in a general working-age population in Southern Germany.

## Methods

### Study design

This study uses data from a standardized lung function test (performed in the F4 sub-cohort) and investigates the relationship between lung function and HRQL which was measured simultaneously, seven years before (S4 cohort) and three years after (F4L cohort) the lung function test.

### Study population

**Figure 1:**
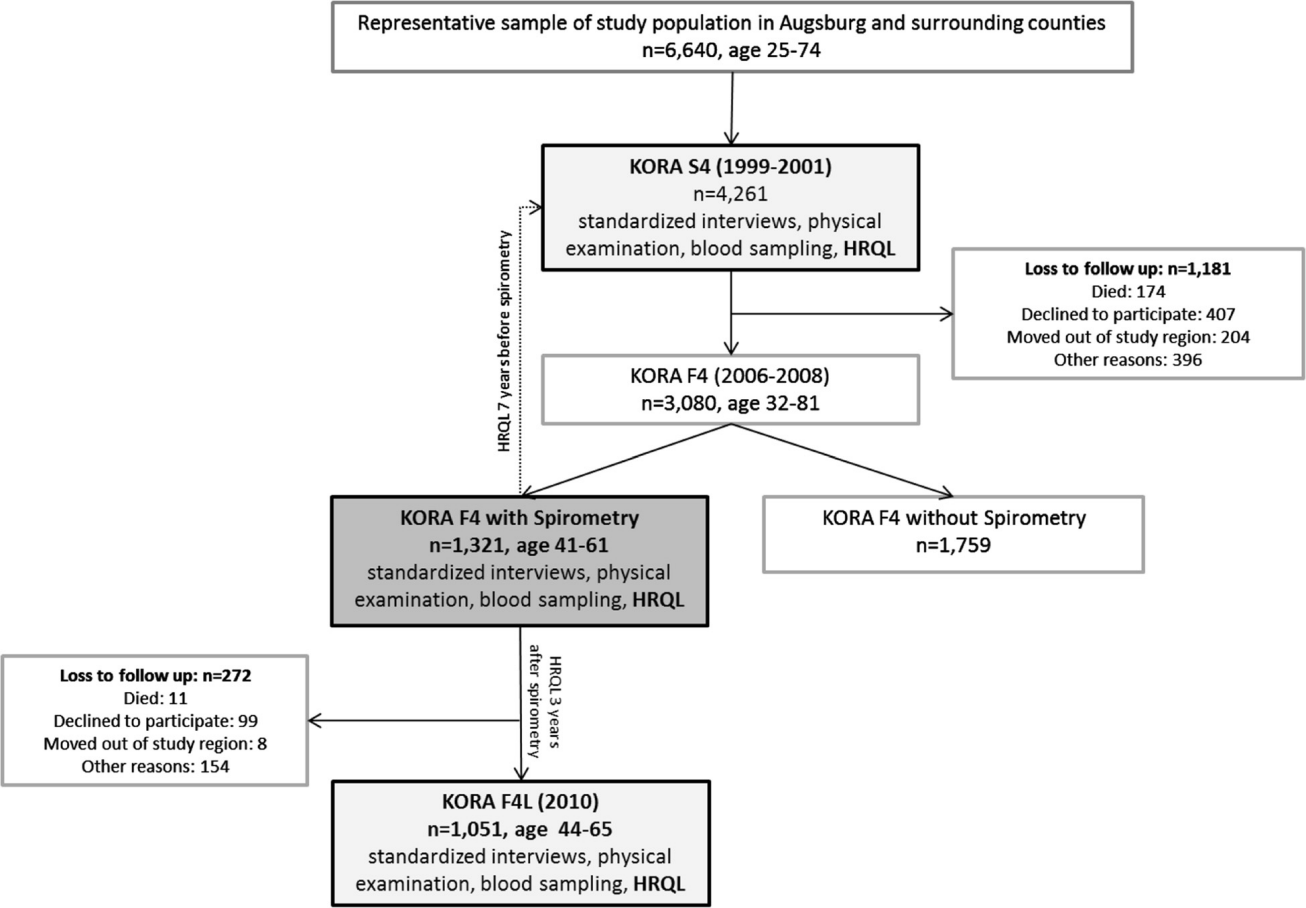
Flow diagram.

The present study was based on data from the population-based KORA (“Cooperative Health Research in the Augsburg Region”) research, a platform for health surveys and subsequent follow-up studies in the fields of epidemiology, health economics and health services research in Southern Germany. Data from the S4 study from the years 1999 to 2001 and its two follow-up studies F4 (2006–2008) and F4L (2010) were used covering an observation period for HRQL of 10 years.The KORA S4 study randomly selected a representative sample of 6,640 adults of German nationality aged 25–74 years in the city of Augsburg and the two adjacent administrative districts from population registries. Of those, 4,261 participated in the baseline examination S4. In the first follow-up examination F4 3,080 participants (72%) were re-examined after seven years. In the F4 follow-up, a subsample (n = 1,321) aged 41–61 years as per reference date (35–54 years at baseline) performed standardized spirometry. Three years later, the F4L study re-examined 79% (n = 1,051) of this subsample (Figure 1).

In all KORA studies, participants performed standardized computer-assisted interviews, questionnaires and examinations. Details about study design, sampling method, data collection and response rates have been published elsewhere [16]. All studies were approved by the responsible ethics committee of the Bavarian Medical Association and written informed consent was obtained from all participants.

### Lung function measurements and COPD definition

In the first follow-up study (F4), standardized spirometry was performed using a pneumotachograph-type spirometer (Masterscreen PC, CardinalHealth, Germany). The spirometer was calibrated at least once a day before measurements, and additionally, an internal control was used to ensure constant instrumental conditions. In order to obtain a minimum of two acceptable and reproducible values, participants performed at least three forced expiratory lung function manoeuvers. Spirometric measurements were in line with the ATS guidelines. Airflow limitation was defined as a pre-bronchodilator FEV_1_/FVC below the lower limit of normal (LLN). Predicted values were calculated from reference equations provided by the Global Lung Function Initiative [17]. Participants with a FEV_1_/FVC ratio ≥ LLN were defined as the non-COPD group. For sensitivity analyses, airflow limitation was defined as the pre-bronchodilator FEV_1_/FVC < 0.7 following modified GOLD criteria [18].

In both definitions, those classified as having airflow limitation were sub-classified as grade 1 with FEV_1_% pred. ≥ 80, grade 2 with 50 ≤ FEV_1_% pred. < 80, grade 3 with 30 ≤ FEV_1_% pred. < 50 and grade 4 with FEV_1_% pred. < 30. Only 1 participant showed grade 3 airflow limitation and was grouped into grade 2+ for analysis. There were no observations in grade 4.

### Health-related quality of life

HRQL information was collected in all three visits, i.e. over a ten year period lasting from 7 years before the lung function test to 3 years after the lung function test. HRQL was assessed using the generic Short Form 12 Health Survey Questionnaire (SF-12) which consists of 12 items selected from the SF-36 [19]. The questionnaire has well-documented reliability and validity and provides the physical and mental health summaries (PCS-12, MCS-12) as two summary scores [20]. The scores yield a mean score of 50 and a standard deviation of 10 in the general U.S. population with higher values indicating better health. A HRQL difference of about three points can be seen as clinically relevant [21].

Participants self-administered the SF-12 questionnaire in the F4 and F4L studies at the study center and completeness was checked by the study personnel. In the S4 study, the SF-12 questionnaire was integrated into the examination interview.

### Co-variables

In standardized computer-assisted face-to-face interviews and in additionally administered questionnaires, information on age, sex, school education (basic (≤9 years), secondary (10–11 years) and higher (≥12 years)), smoking status (current, former and never smoker (self-reports were compared with previous or following information on smoking status)), and self-reported data on the presence of a number of comorbidities were collected.

The following comorbid conditions were included as binary covariates as they have been shown to be associated with COPD [3,4] and may influence HRQL [22]: heart failure, myocardial infarction, stroke, cancer and diabetes.

For cancer and myocardial infarction, participants were asked if they were ever diagnosed with these diseases by a physician. For heart failure, this question was restricted to the last 12 months. Participants were also asked if they had ever been treated for a stroke in a hospital. Diabetes was defined as a physician-diagnosed history of diabetes or the intake of hypoglycemic drugs according to the ATC code A10. Furthermore, BMI (weight in kilograms/height squared in meters obtained by standardized medical examination by trained medical staff) was included in order to account for possible weight differences. Finally, participants were asked if they had ever had a physician diagnosis of chronic bronchitis, COPD or emphysema.

### Statistical analyses

Of 1,321 participants with complete lung function data 1,291 had complete HRQL data at the time of the lung function test (F4) and in the previous study S4 and 1,017 three years later at F4L. For participants with missing information on education (n = 3), the most frequent category (low education) was assumed, and for three participants with missing information on BMI, BMI data from the previous or following examination was assumed. Five participants had missing information on heart failure and were excluded from the extended analysis considering comorbidities.

Characteristics of participants in different COPD grades and without COPD in the F4 examination as well as unadjusted values for PCS-12 and MCS-12 were compared using analysis of variance (ANOVA) for continuous variables and Chi^2^-tests or Fisher’s exact tests for categorical variables.

In order to estimate the effect of airflow limitation on past, present and future HRQL, regression analyses with HRQL as the dependent variable were performed using linear mixed models with random intercepts. These models are a repeated measurement method that can deal with different numbers of observations per subject. They do not exclude patients with missing S4 or F4L data but use the available information before drop-out to estimate means and covariances. By accounting for within-patient correlations, appropriate adjustments for parameter estimates are made at times when data are incomplete [23].

Regression models were adjusted for age, sex and school education in basic models and in addition for smoking status, five selected comorbidities and BMI in extended models. Comorbidities, smoking status and BMI were time-varying while COPD grade, age at F4, sex and education were constant. The variance of the random intercept distribution was used to assess heterogeneity of mean HRQL levels between subjects.

Analyses were performed using the SAS software (SAS Institute Inc., Cary, NC, USA, Version 9.3) package. P-values of 0.05 or less were considered statistically significant.

### Sensitivity analyses

For sensitivity analyses, FEV_1_ and the FEV_1_/FVC ratio as well as their interactions with time were considered as continuous variables instead of the COPD grade.

In order to investigate a potential misclassification, additional sensitivity analyses were performed excluding asthmatic subjects from the COPD group. First, subjects who showed an airflow limitation without having a physician diagnosis of COPD but who reported at the time of the lung function test an asthma attack in the last 12 months (n = 4) were excluded. Second, subjects with airflow limitation who reported a history of asthma but no physician diagnosis of COPD (n = 15) were excluded. Furthermore, a previous history of asthma was considered in the extended model as a further comorbidity.

## Results

In all, 101 or 7.8% of the 1,291 participants with valid lung function test and complete HRQL data were classified as having an airflow limitation in the F4 study. Of these, 59.4% were in grade 1. The majority of COPD cases found by spirometry were undiagnosed before: 20% of participants with COPD grade 1 reported a physician diagnosis of chronic bronchitis, COPD or emphysema and 45% of the grade 2+ group.

Characteristics of subjects at the time of the lung function test are shown in Table 1. Groups with different grades of airflow limitation differed significantly regarding age and smoking status, while there were no differences as to sex, educational level, BMI and comorbidities.

**Table 1:** Characteristics of participants at the time of lung function test (F4 study)

	**Total**	**No COPD**	**COPD grade 1**	**COPD grade 2+**	**p-value**
	n = 1321	n = 1220	n = 60	n = 41	
Age (years)	51.6 (5.7)	51.6 (5.7)	50.0 (5.7)	52.9 (6.3)	0.04^a^
Female sex	703 (53.2%)	648 (53.1%)	34 (56.7%)	21 (51.2%)	0.83^b^
Educational level:					
Basic	635 (48.1%)	579 (47.5%)	30 (50.0%)	26 (63.4%)	0.16^b^
Secondary	343 (26.0%)	318 (26.1%)	14 (23.3%)	11 (26.8%)	
High	343 (26.0%)	323 (26.5%)	16 (26.7%)	4 (9.8%)	
Smoking status:					
Never smoker	477 (36.1%)	462 (37.9%)	10 (16.7%)	5 (12.2%)	<0.0001^b^
Current smoker	306 (23.2%)	264 (21.6%)	23 (38.3%)	19 (46.3%)	
Former smoker	538 (40.7%)	494 (40.5%)	27 (45.0%)	17 (41.5%)	
Body mass index (kg/m^2^):					
Normal (18.5 ≤ BMI < 25)	455 (34.4%)	410 (33.6%)	31 (51.7%)	14 (34.2%)	0.11^c^
Underweight (BMI < 18.5)	4 (0.3%)	4 (0.3%)	0 (0.0%)	0 (0.0%)	
Overweight (25 ≤ BMI < 30)	520 (39.4%)	482 (39.5%)	21 (35.0%)	17 (41.5%)	
Obese (BMI > 30)	342 (25.9%)	324 (26.6%)	8 (13.3%)	10 (24.4%)	
Comorbid conditions:					
Heart failure (nmiss = 7)	15 (1.1%)	13 (1.1%)	2 (3.3%)	0 (0.0%)	0.22^c^
Myocardial infarction	22 (1.7%)	22 (1.8%)	0 (0.0%)	0 (0.0%)	0.81^c^
Stroke	14 (1.1%)	13 (1.1%)	1 (1.7%)	0 (0.0%)	0.67^c^
Cancer	62 (4.7%)	57 (4.7%)	5 (8.3%)	0 (0.0%)	0.16^c^
Diabetes	50 (3.8%)	48 (3.9%)	2 (3.3%)	0 (0.0%)	0.57^c^

Data are mean (standard deviation) or n (%).

^a^based on ANOVA ^b^based on Chi^2^-test ^c^based on Fishers exact test.

Without adjusting for covariates, participants with COPD grade 1 or 2+ showed lower physical scores (PCS-12) than the non-COPD group (47.8/44.2 compared to 48.8 in controls, p = 0.004) at the time of spirometry (Table 2). This difference was also apparent seven years before and three years after spirometry. The physical score decreased slightly over time in all COPD groups as in controls. No group differences were observed with respect to the mental SF-12 score (MCS-12) neither at the time of spirometry nor in the previous (S4-study) or following study (F4L-study).

**Table 2:** Unadjusted analysis of the physical and mental component score (PCS-12 and MCS-12) of the SF-12 at time of spirometry, seven years before and three years later

	**Total**	**No COPD**	**COPD grade 1**	**COPD grade 2+**	**p-value**^**a**^
**PCS-12**					
S4 (−7 years)	48.87 (8.22)	49.05 (8.03)	48.09 (8.50)	44.84 (11.85)	0.002
F4 (baseline)	48.57 (8.66)	48.75 (8.52)	47.83 (9.60)	44.23 (10.30)	0.004
F4L (+3 years)	48.45 (8.64)	48.59 (8.61)	47.88 (8.79)	44.16 (8.74)	0.029
**MCS-12**					
S4 (−7 years)	50.65 (9.14)	50.63 (9.13)	50.24 (8.37)	51.83 (10.66)	0.607
F4 (baseline)	50.72 (9.22)	50.70 (9.25)	50.44 (8.81)	51.98 (9.02)	0.672
F4L (+3 years)	51.57 (9.32)	51.61 (9.26)	50.75 (9.59)	51.40 (11.21)	0.799

PCS-12: Physical Component Score, MCS-12: Mental Component Score.

^a^based on ANOVA.

**Figure 2:**
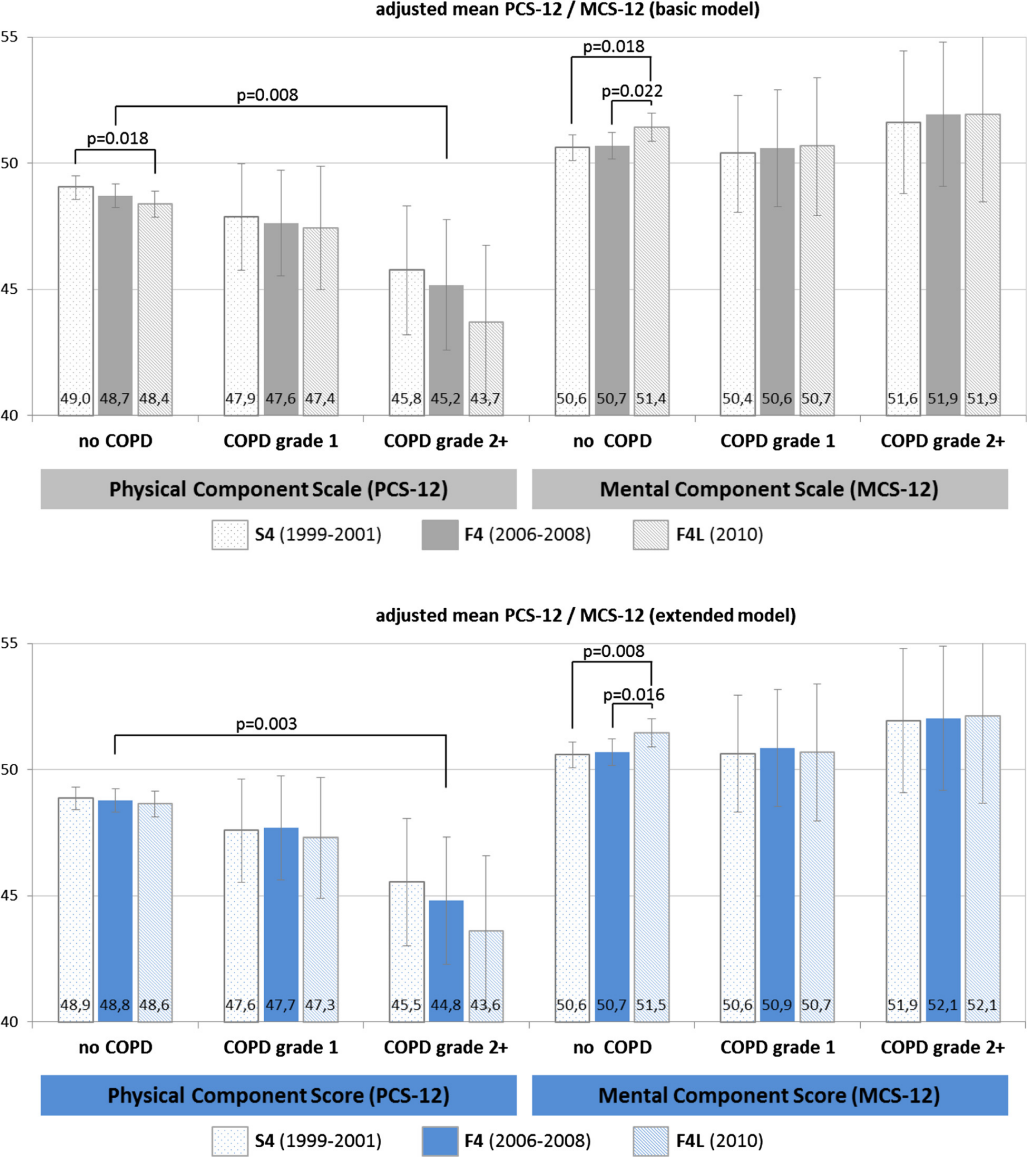
Adjusted mean PCS-12 and MCS-12 scores at S4, F4, F4L.

Linear mixed models showed a significant negative effect of COPD grade 2+ on PCS-12 of −3.5 points (p = 0.008) at the time of spirometry (Table 3). This effect persisted (−4.0 points, p = 0.003) when comorbidities were considered in the extended model. There was a small, but insignificant negative effect of COPD grade 1 in both models (−1.1/-1.1 points). Interaction terms of COPD and time, representing the longitudinal effect of COPD on PCS-12 were not significant, both in the basic and in the extended model. In the extended model, the dimension of the negative association of obesity, heart failure, myocardial infarction, stroke and cancer with PCS-12 was similar to the effect of COPD grade 2+. Regarding MCS-12, no significant associations with COPD were found.Resulting adjusted mean PCS-12 and MCS-12 scores are shown in Figure 2. At the time of spirometry (F4), adjusted mean PCS-12 scores were 47.6/45.2 for the COPD grade 1/grade 2+ group and 48.7 for participants without airflow limitation. For MCS-12, adjusted mean scores were 50.6/51.9 and 50.7, respectively.

**Table 3:** Regression analysis: linear mixed models

**Effect**	**Physical component: PCS-12**		**Mental component: MCS-12**	
	**Basic model**	**Extended model**	**Basic model**	**Extended model**
No COPD	ref.	ref.	ref.	ref.
COPD grade 1	−1.10	−1.10	−0.10	0.16
COPD grade 2+	−3.54**	−3.98**	1.24	1.36
Time (1, F4L)*no COPD	ref.	ref.	ref.	ref.
Time (1, F4L)*COPD grade 1	0.15	−0.26	−0.66	−0.94
Time (1, F4L)*COPD grade 2+	−1.16	−1.08	−0.75	−0.71
Time (−1, S4)*no COPD	ref.	ref.	ref.	ref.
Time (−1, S4)*COPD grade 1	−0.06	−0.18	−0.12	−0.12
Time (−1, S4)*COPD grade 2+	0.27	0.65	−0.23	−0.02
Time (0, F4)	ref.	ref.	ref.	ref.
Time (−1, S4)	0.32	0.08	−0.08	−0.09
Time (1, F4L)	−0.33	−0.15	0.73*	0.77*
Age 41–45 yrs.	ref.	ref.	ref.	ref.
Age 46–50 yrs.	0.48	0.51	0.83	0.81
Age 51–55 yrs.	−1.04	−0.62	0.27	0.29
Age ≥ 56 yrs.	−2.94***	−2.42***	0.66	0.68
Sex: male	ref.	ref.	ref.	ref.
Sex: female	−0.78*	−1.00**	−2.33***	−2.48***
Educational level:				
Basic education	ref.	ref.	ref.	ref.
Secondary education	1.61***	1.20**	−0.66	−0.70
High education	2.40***	1.85***	−0.29	−0.44
Smoking status:				
Never smoker		ref.		ref.
Current smoker		−0.82*		−1.05*
Former smoker		−0.92		−0.56
BMI:				
Normal (18.5 ≤ BMI < 25)		ref.		ref.
Underweight (BMI < 18.5)		−0.60		−1.75
Overweight (25 ≤ BMI < 30)		−0.48		−0.34
Obese (BMI > 30)		−2.48***		0.25
Comorbidities:				
Heart failure		−4.94***		−2.75*
Myocardial infarction		−3.33*		0.77
Stroke		−5.58***		−4.03*
Cancer		−3.21***		0.28
Diabetes		−1.72*		−2.05*
Intercept	49.12	50.64	51.71	52.26

*p < 0.05, **p < 0.01, ***p < 0.001.

S4: −7 years, F4: baseline, F4L: +3 years.

Mean PCS-12 scores for the COPD grade 2+ group were significantly (p < 0.05) reduced in every study (−3.5 in F4, −3.3 in S4 and −4.7 in F4L) compared with the group without airflow limitation. This difference persisted even when comorbidities were considered in the extended model.

Regarding longitudinal changes over time, a small decline in the physical score was observed in all groups. The decline over ten years was significant in the reference group, but not in the COPD grade 1 and 2+ groups despite being larger. Regarding mental HRQL aspects, the ten year increase in MCS-12 was significant in the group without airflow limitation, but not in the COPD groups. Consideration of comorbidities hardly changed the adjusted mean PCS-12 and MCS-12.

The variance of random intercepts ranged from 27.5 (SE 1.7) to 31.7 (SE 2.1), indicating a high degree of heterogeneity in mean HRQL levels between individuals.

Using the GOLD definition as an alternative approach to define COPD resulted in a COPD prevalence of 10% (n = 132), with 62.9% of cases classified as grade 1. PCS-12 estimates for COPD grade 2+ were smaller than according to the LLN definition. Estimates for COPD grade 1 hardly changed and were still insignificant. MCS-12 estimates as well as longitudinal associations remained insignificant (Additional file 1: Appendix Table A1-A4).

### Sensitivity analyses

When considering FEV_1_% pred. as a continuous variable instead of the COPD groups, there was a significant positive effect of FEV_1_% pred. on PCS-12 (β = 0.077, p < 0.001), but not on MCS-12. All interaction terms between FEV_1_% pred. and time (S4, F4, F4L) were insignificant. When considering the FEV_1_/FVC ratio as a continuous variable, the effect on PCS-12 was insignificant (β = 0.072, p = 0.057). Neither the effect on MCS-12 nor the interaction terms with time were significant.

The proportion of participants reporting a history of asthma is larger in the COPD group (30.7%) than in controls (7.5%). Nevertheless, a high proportion of COPD cases reporting a previous history of asthma also report a physician diagnosis of COPD (51.6%). Only 4 COPD cases reported no physician diagnosis of COPD but asthma symptoms in the last 12 months. Excluding these subjects did not change parameter estimates and adjusted PCS-12 and MCS-12 means. Excluding 15 subjects who reported a previous history of asthma and no physician diagnosis of COPD increased the cross-sectional effect of COPD grade 2+ from 3.5 to 5.2 (p = 0.001), but did not change the effect of COPD grade 1 or the insignificant longitudinal effects of COPD.

Considering a previous history of asthma in the extended regression models slightly reduced the effect of COPD grade 2+ on PCS-12 from −4.0 to −3.5 points but did not change estimates for MCS-12.

## Discussion

The objective of this study was to examine time trends in HRQL over ten years of middle-aged persons with early COPD stages compared to controls without airflow limitation and to quantify the effect of COPD under consideration of common COPD-related comorbidities.

We found a clinically relevant, negative cross-sectional association of COPD grade 2+ with the physical aspects of HRQL, but no significant effects of grade 1. The effect of grade 2+ was not confounded by common COPD comorbidities. In the longitudinal perspective of ten years there were no significant effects of COPD on HRQL. A somewhat more pronounced decrease in physical health over 10 years was observed in participants with COPD grade 2+, but this decrease was not statistically significant. Regarding mental aspects of HRQL, we found a slight increase over time for all participants. This increase was smaller in both COPD groups compared to the group without airflow limitation. Although all longitudinal changes found here were small, results indicate that disease progression from early to higher COPD grades implies loss in HRQL. Therefore, efficient interventions that slow disease progression, such as smoking cessation approaches, are not only clinically but also from the perspective of quality of life very reasonable at early stages of disease [24,25]. Our results are in accordance with previous studies which found a negative relationship between HRQL and COPD which increases with disease severity [12,26-30]. A number of them were also performed in a general population setting and used the generic SF-12 or SF-36 instrument for HRQL measurements [12,26,27].

Based on data from the large international Burden of Obstructive Lung Disease (BOLD) study, a recent cross-sectional analysis by Janson et al. investigated the impact of COPD on health status as measured by the SF-12 [12]. The population-based setting of this epidemiologic study was comparable to our analysis. Estimates for physical health found in this study were very similar to our results: Grade 2 COPD according to the LLN definition was associated with a reduction of −3.2 points of PCS-12 while estimates for grade 1 were not significant. However, BOLD reported significant negative associations between COPD grade 2 and higher and the mental SF-12 score which could not be confirmed in our study. Similar to our analysis, effect estimates were slightly stronger using the LLN definition compared with the GOLD staging (−3.2 vs. -3.0 for grade 2). The associations between COPD and HRQL found by Janson et al. persisted independent of four common comorbidities (heart disease, hypertension, diabetes and stroke). In our extended analysis considering similar comorbidities, the effect of COPD grade 2+ was also significant and of similar size to the basic model without comorbidities.

A population-based study from Norway also reported a negative association of COPD with SF-12 physical scores [26]. Grade 2 was associated with a −3.1 reduction of PCS-12, while estimates for grade 1 and regarding MSC-12 were not significant. DiBonventure et al. described negative effects of COPD on both MCS-12 (−1.3) and PCS-12 (−6.9) in a population-based setting [29]. As this analysis used self-reported information on COPD diagnosis without lung function measurement the reason for stronger effects on HRQL may be the fact that undiagnosed COPD patients were not considered.

Bridevaux et al. also focused on early COPD stages identified in a population-based sample and found that subjects in COPD stage 1 with respiratory symptoms had lower PCS scores than the reference category while asymptomatic subjects with stage 1 COPD showed similar PCS scores to the reference group [31]. For MCS, no differences were observed for COPD in stage 1.

Regarding common COPD-related comorbidities, only a few studies focused on the relationship of COPD and HRQL in COPD patients while considering comorbid conditions.

In a study on previously diagnosed COPD patients using the SF-36, van Manen et al. reported that impairments in physical functioning, in role functioning due to physical problems and in vitality were related both to COPD and comorbidity, whereas social functioning, mental health and role functioning due to emotional problems were unrelated to COPD [32]. However, comorbidity was considered as a dummy variable together with an interaction with COPD. Therefore, the individual effect of single comorbid conditions was not provided.

Burgel et al. found in a clinical cohort of stable COPD patients that dyspnea, exacerbations and depression were important determinants of disease-specific HRQL while the degree of airflow limitation, low BMI and coronary arterial disease had only a modest impact and other cardiovascular conditions, obesity and diabetes showed no significant impact on HRQL [22]. However, as this study used a disease-specific HRQL instrument some effects of comorbid conditions on HRQL may not have been captured.

Only a few studies have investigated COPD-related changes in HRQL over time. Most of these studies focused on HRQL changes of hospitalized patients (e.g. during acute exacerbations [33,34]), newly detected COPD cases [35], patients in the end-stage setting [15] or observed HRQL changes after pharmacological and non-pharmacological interventions [13,14]. In general, weak associations between changes in lung function and HRQL developments were found [36,37].

Koskela et al. assessed HRQL as measured by the generic 15D questionnaire over 4 years in elderly COPD patients recruited from two university hospitals in Finland and found that the majority of them showed declining HRQL over time. A clinically relevant deterioration was observed within 1.7 years in this patient group [38]. Marin et al. observed changes in health status as measured by the disease-specific SGRQ in a cohort of COPD patients over ten years and found that SGRQ scores worsen with increasing COPD severity [39]. Oga et al. examined both the course of disease specific HRQL and of lung function in stable COPD patients within three years [36]. They observed a small decline in HRQL over time, but found that changes in health status were not correlated with changes in lung function.

As these previous longitudinal studies use data from clinical COPD patients without comparison to controls without airflow limitation, their results are not directly transferable to COPD patients found in a younger general population sample with a high percentage of undiagnosed cases.

One strength of our study is that it is based on data from a general population cohort and therefore may yield results for early stages of COPD that are more generalizable than those from clinical cohorts. Although patient recruitment from physicians or clinics can be expected to result in an unbiased sample for those with severe or very severe COPD, mild or moderate disease stages which are often undiagnosed, are commonly underrepresented [40]. However, it is important to address early stages of COPD as confirmed in a previous population-based study where patients with undiagnosed COPD also showed impaired HRQL compared to participants without COPD [41]. Furthermore, the longitudinal character of our analysis allowed us to address the scarcely investigated question of HRQL changes over time in early disease stages.

One limitation of the study is a possible selection bias. As it has to be expected that non-participants of all three surveys used are more impaired than participants, the study sample may not be representative for Germany. Although this bias is likely to be less pronounced in the early disease stages we focus on, this problem can still affect both COPD patients and controls. Participants who were lost to follow-up between F4 and F4L were significantly more frequently smokers or former smokers, showed more often airflow limitation and had a lower PCS-12 than participants who took part in both studies. The main reason of drop-out was refusal of participation while death and moving out of the study region were of minor importance. Selection effects could result in both over- and underestimation of the impact of COPD, which limits the generalizability of our findings. These effects could also be a reason for the weak longitudinal effects found in our study which do not reflect disease progression over time.

Another potential problem is the limited number of COPD patients in the sample. The prevalence of COPD found in our study (7.8% according to LLN/10.0% according to the GOLD definition) is slightly lower than the BOLD estimates for Germany [11]. But comparability is limited as the BOLD study also included higher age groups which have a higher COPD prevalence. A similar population-based study in northern Germany (SHIP) also reported a lower COPD prevalence of 7.2% (defined by the LLN criteria, post-bronchodilator spirometry) but included age groups from 35 to 90 years [42].

In general, there is a large range of COPD prevalence data in international studies [43,44]. Population-based studies such as NHANES reported a COPD prevalence of 14.0% in a U.S. population of 40–59 years [45] while Maio et al. reported a multinational COPD prevalence of 10.5% in 40–49 year olds and 12.0% in 50–59 year olds [46] (both studies used pre-bronchodilator spirometry and LLN definition). However, differences in demographics, smoking behavior or diagnostic approaches have to be considered when directly comparing COPD prevalences.

The limited number of COPD cases in our study, especially of cases in higher COPD grades, may result in a low power and failure to detect differences e.g. in the mental SF-12 score. However, it is also possible that there really is no difference in MCS-12 in early stages. This is supported by other studies that also found differences in PCS, but not in MCS scores either between GOLD stages [27] or between COPD patients in early stages and controls [26,30].

Although the asthma and COPD overlap is widely acknowledged, pre-bronchodilator spirometry may misclassify asthmatic subjects as having COPD [47]. Nevertheless, for simplicity reasons, pre-bronchodilator spirometry is often performed and accepted in population-based studies [48]. We considered the possibility of misclassification in sensitivity analyses but our results barely changed. The proportion of never smokers in the groups with airflow limitation is also comparable to or below that reported in other studies [49,50].

Furthermore, the presence of comorbid conditions was based on self-report, which may limit the validity of these data. We could not find increased comorbidity in COPD cases as described in previous studies [3,4]. This may be due to the fact that a younger population in early COPD stages was considered in our analysis while relevant comorbidities may develop predominantly later with age and COPD progression.

Finally, as we did not have longitudinal lung function data, further studies are needed to assess the long-term effects of COPD as well as lung function changes over time on HRQL development which also include the whole COPD severity spectrum.

## Conclusion

Despite small HRQL differences between COPD patients in early stages and controls and small changes over a ten year time period, our results indicate that it is important to prevent subjects with airflow limitation from disease progression to more severe grades. Furthermore, awareness of HRQL impairments in early and even in undiagnosed stages is vital for early identification of persons at risk and an efficient therapy at early stages to slow disease progression and, thus, maintain high HRQL in COPD patients for as long as possible.

## Competing interests

None of the authors has any conflicts of interest to disclose.

## Authors’ contributions

MW conceptualized the paper, performed the statistical analysis, interpreted the data and drafted the manuscript. MH assisted in the statistical analysis and in writing the paper. HS, SK, and JH were involved in the coordination of the study; AP was involved in the conception of the study; and all commented on drafts of the paper. RH was involved in the conception of the study and assisted in the conceptualization of the paper and in writing the manuscript. All authors critically reviewed each draft of the manuscript and approved the final manuscript.

## Pre-publication history

The pre-publication history for this paper can be accessed here:

http://www.biomedcentral.com/1471-2466/14/134/prepub

## Supplementary Material

Additional file 1 Appendix Table. Click here for file
