# Supplementary material for: Health-related quality of life and chronic obstructive pulmonary disease in early stages – longitudinal results from the population-based KORA cohort in a working age population
Source: BMC Pulm Med. 2014 Aug 9;14:134. doi: 10.1186/1471-2466-14-134 (PMC4130122; doi:10.1186/1471-2466-14-134)
Supplement: Additional file 1 — Appendix Table. [file 1471-2466-14-134-S1.doc]

**Additional file 1: APPENDIX**

**Table A1: Unadjusted analysis of the Physical and Mental Component Score (PCS-12 and MCS-12) of the SF-12 at time of spirometry, sever years before and three years later: defining COPD according to GOLD definition**

|  | **Total** | **No COPD** | **COPD grade 1** | **COPD grade 2+** | **p-value ^a^** |
| --- | --- | --- | --- | --- | --- |
| n | n=1321 | n=1189 | n=83 | n=49 |  |
| with complete HRQL | n=1291 | n=1161 | n=83 | n=47 |  |
| **PCS-12** | | | | | |
| S4 (-7 years) | 48.87 (8.2) | 49.09 (8.0) | 48.35 (8.4) | 44.50 (11.7) | 0.0002 |
| F4 (baseline) | 48.57 (8.7) | 48.79 (8.5) | 47.91 (9.3) | 44.45 (10.1) | 0.003 |
| F4L (+3 years) | 48.45 (8.6) | 48.64 (8.6) | 47.96 (8.8) | 43.84 (8.8) | 0.006 |

| **MCS-12** | | | | | |
| --- | --- | --- | --- | --- | --- |
| S4 (-7 years) | 50.65 (9.1) | 50.58 (9.2) | 51.00 (8.2) | 51.65 (10.3) | 0.60 |
| F4 (baseline) | 50.72 (9.2) | 50.69 (9.3) | 50.41 (9.2) | 51.97 (8.5) | 0.62 |
| F4L (+3 years) | 51.57 (9.3) | 51.61 (9.3) | 51.09 (9.5) | 51.26 (10.4) | 0.85 |

PCS-12: Physical Component Score, MCS-12: Mental Component Score
^a^ based on ANOVA

**Table A2: Regression analysis: Linear mixed models: COPD according to GOLD definition**

| effect | **Physical Component: PCS-12** | | | | **Mental Component: MCS-12** | | | |
| --- | --- | --- | --- | --- | --- | --- | --- | --- |
|  | **basic model** | | **extended model** | | **basic model** | | **extended model** | |
| no COPD | ref. |  | ref. |  | ref. |  | ref. |  |
| COPD grade 1 | -0.77 |  | -0.83 |  | -0.37 |  | -0.11 |  |
| COPD grade 2+ | -3.33 | ** | -3.69 | ** | 1.03 |  | 1.13 |  |
| time(1)*no COPD | ref. |  | ref. |  | ref. |  | ref. |  |
| time(1)*COPD grade 1 | 0.13 |  | -0.05 |  | -0.22 |  | -0.44 |  |
| time(1)*COPD grade 2+ | -1.14 |  | -1.24 |  | -0.78 |  | -0.72 |  |
| time(-1)*no COPD | ref. |  | ref. |  | ref. |  | ref. |  |
| time(-1)*COPD grade 1 | -0.10 |  | -0.16 |  | 0.59 |  | 0.57 |  |
| time(-1)*COPD grade 2+ | -0.32 |  | -0.05 |  | -0.33 |  | -0.12 |  |

* p<0.05, ** p<0.01, *** p<0.001

**Table A3: Adjusted means of PCS-12 and MCS-12 (basic model, GOLD definition)**

|  | **No COPD** | **COPD grade 1** | **COPD grade 2+** |
| --- | --- | --- | --- |
| **PCS-12** | | | |
| S4 (-7 years) | 49.07 | 48.20 | 45.42 |
| F4 (baseline) | 48.73 | 47.96 | 45.40 |
| F4L (+3 years) | 48.41 | 47.77 | 43.94 |
| **MCS-12** | | | |
| S4 (-7 years) | 50.59 | 50.82 | 51.30 |
| F4 (baseline) | 50.72 | 50.35 | 51.75 |
| F4L (+3 years) | 51.44 | 50.85 | 51.69 |

**Table A4: Adjusted means of PCS-12 and MCS-12 (extended model, GOLD definition)**

|  | **No COPD** | **COPD grade 1** | **COPD grade 2+** |
| --- | --- | --- | --- |
| **PCS-12** | | | |
| S4 (-7 years) | 48.90 | 47.90 | 45.16 |
| F4 (baseline) | 48.80 | 47.97 | 45.11 |
| F4L (+3 years) | 48.66 | 47.77 | 43.74 |
| **MCS-12** | | | |
| S4 (-7 years) | 50.57 | 51.02 | 51.58 |
| F4 (baseline) | 50.70 | 50.59 | 51.83 |
| F4L (+3 years) | 51.46 | 50.91 | 51.87 |
